# P32 Assessing outpatient parenteral antimicrobial therapy (OPAT) referrals to determine number of bed days saved in secondary care

**DOI:** 10.1093/jacamr/dlad066.036

**Published:** 2023-06-14

**Authors:** Teresa Chan, Daniel Hearsey

**Affiliations:** Royal Cornwall Hospitals Trust (RCHT), UK; Royal Cornwall Hospitals Trust (RCHT), UK

## Abstract

**Background:**

Outpatient parenteral antimicrobial therapy (OPAT) is a safe and effective service to manage infections in patients.^1^ OPAT allows medical admission avoidance for stable patients and allows for early discharge for patients with ongoing IV antimicrobial therapy needs. OPAT therefore allows the optimization of hospital bed management and relieve pressures on the NHS.^2^ The primary aim of this audit is to determine the number of bed days saved in secondary care through early discharge and admission avoidance by the OPAT service which is completed by Cornwall Foundation Trust’s Acute Care at Home (ACAH) Team. This study did not require ethics approval.

**Objectives:**

(i) To quantify the total number of bed days saved by referral to OPAT; (ii) to determine the total number of days of each IV antibiotic received by patients under the OPAT service and (iii) to compare treatment course lengths for infections being managed by the OPAT service against nationally reported data.

**Standards:**

100% of patients must be receiving IV antibiotics with an indication whilst under OPAT. 100% of patients must have an IV antibiotic dose, frequency and administration method documented. 100% of patients must have an acceptance date by the OPAT team.

**Methods:**

Patients were identified from daily handover documents from the ACAH team for the period of 1 February to 28 February 2023. Data collection was obtained using the clinical recording and electronic health recording system, RiO and Maxims, and the electronic prescribing and medicine administration (EPMA) system. The number of bed days saved is calculated from the acceptance date to OPAT to the discharge date from OPAT for each patient.

**Results:**

A total of 70 patients were captured during initial data gathering, of which 41 patients met the inclusion criteria for the audit. Of these 41 patients meeting the audit criteria, 21 (51.2%) patients were referred from primary care and 20 (48.9%) patients were referred from RCHT. The total sum of bed days saved was 343 days, and the total sum of OPAT treatment days was 302 days. The most prescribed antibiotic was Ceftriaxone, with 27 patients (%) receiving this antibiotic; other antibiotics included the following: Tazocin (*n* = 5), ertapenem (*n* = 5), flucloxacillin (*n* = 2), gentamicin (*n* = 1), meropenem (*n* = 1) and teicoplanin (*n* = 1). The recorded indications were recorded, and when compared with the average treatment duration for each infection type against the NORS 2015–2020 data,^3^ the average treatment days in this audit were within range.

**Conclusions:**

These results demonstrate that the OPAT service utilized by RCHT has a significant impact on bed utilization, through reducing hospital admissions and facilitating early discharge. Course lengths are in keeping with the national reported average indicating that patients are receiving comparable course lengths to other OPAT providers; further work to compare these course lengths against national best practice would be useful. There are limitations to the results, as the number of bed days saved was capped to the end of the audit data collection period. Recommendation to improve would be to re-audit with a longer data collection time frame.
